# Health-related quality of life is strongly associated with self-efficacy, self-esteem, loneliness, and stress in 14–15-year-old adolescents: a cross-sectional study

**DOI:** 10.1186/s12955-020-01585-9

**Published:** 2020-11-02

**Authors:** Hilde Timenes Mikkelsen, Kristin Haraldstad, Sølvi Helseth, Siv Skarstein, Milada Cvancarova Småstuen, Gudrun Rohde

**Affiliations:** 1 Department of Health and Nursing, Faculty of Health and Sport Sciences, University of Agder, Postbox 422, 4604 Kristiansand, Norway; 2 Department of Nursing and Health Promotion, Faculty of Health Sciences, Oslo Metropolitan University, Oslo, Norway; 3 Department of Clinical Research, Sorlandet Hospital, Kristiansand, Norway

**Keywords:** Health-related quality of life, Adolescents, Self-efficacy, Self-esteem, Loneliness, Stress

## Abstract

**Background:**

To enhance and better understand health-related quality of life (HRQOL) in adolescents, it is important to study factors associated with HRQOL. The present study aimed to assess possible associations between sociodemographic variables, self-efficacy, self-esteem, pain, sleep, loneliness, stress and HRQOL in 14 to 15-year-old adolescents.

**Methods:**

A cross-sectional study was performed among 696 adolescents (14–15 years) in a school-based setting. Sociodemographic variables, self-efficacy, self-esteem, pain, sleep, loneliness and stress were analyzed. The variables were all assessed with well-validated instruments. HRQOL was analyzed using KIDSCREEN 27. Analyses included Chi-square, independent t-tests, Mann–Whitney U tests, linear regression analyses and hierarchical regression analyses. The results from linear regression models were expressed as standardized beta.

**Results:**

The adolescents generally reported high levels of HRQOL. However, girls scored significantly worse on HRQOL, self-efficacy, self-esteem, pain, sleep, loneliness and stress compared to boys. Using hierarchical regression analyses we found that Self-efficacy (beta = 0.11–0.24), Self-esteem: (beta = 0.12–0.21), Loneliness: (beta = − 0.24 to − 0.45) and Stress: (beta = − 0.26 to − 0.34) revealed the strongest associations with the HRQOL dimensions. Sociodemographic-, pain- and sleep related covariates were all significantly associated with some of the KIDSCREEN subscales, however their effect on the outcome was smaller than for the psychosocial variables listed above. Being a girl, not living with both parents, not having both parents working, being absent from school more than 4 days, having pain and having lack of enough sleep were all independently negatively associated with HRQOL.

**Conclusions:**

HRQOL is strongly associated with self-efficacy, self-esteem, loneliness and stress in 14 to 15-year-old adolescents. Our findings indicate that positive psychosocial factors such as self-efficacy and self-esteem might play a buffer role for negative psychosocial factors (e.g. stress) in adolescents. Further, our results show that girls score significantly worse on factors that are associated to HRQOL compared to boys. To improve HRQOL in school-based populations of adolescents, we suggest that future interventions should aim to strengthen self-efficacy and self-esteem. We recommend gender specific interventions.

## Introduction

Quality of life (QOL) is an important concept and target for research and practice in the fields of health and medicine [1]. The term “health-related quality of life” (HRQOL) is a multidimensional construct that includes the individual’s subjective perspectives on the physical, psychological, social, and functional aspects of health [2]. The World Health Organization (WHO) emphasizes well-being and HRQOL as goals for public health, especially among adolescents, and underlines the need for research to identify the key determinants for health problems in this age group [3]. Moreover, WHO notes that adolescent health and well‐being are essential for healthier and more sustainable societies [4, 5].

Adolescence is a life phase between childhood and adulthood in which the opportunities for health are great and where future patterns of adult health are established [4–7]. It is also a vulnerable period in life, and can be challenging with respect to independence from caregivers, increase of autonomy and social role transitions [5, 6, 8–11]. Although the vast majority of Norwegian adolescents are content with their lives and generally report good health [16], an increasing number of adolescents in Norway and other countries report psychosocial problems and health complaints in everyday life such as loneliness, stress, insufficient and poor-quality sleep, pain and high intake of over-the-counter analgesics (OTC analgesics) [9, 11–20], indicating a need for continued efforts in health promotion among adolescents.

Research has identified variables associated with HRQOL such as gender and age. HRQOL often declines during adolescence, and girls tend to report lower HRQOL than boys [16, 21–27]. Family, parents, and siblings are important for adolescents’ HRQOL [8, 15, 28], and HRQOL have been observed to be generally lower in those with low socioeconomic status (SES) and poor social support [29–31]. Furthermore, studies have shown that positive psychosocial factors such as self-efficacy and self-esteem have a positive impact on HRQOL in adolescence [22, 25, 31–34], while health-related and negative psychosocial factors such as stress, pain, high intake of OTC analgesics, loneliness, school absenteeism and insufficient and poor-quality sleep are associated with lower HRQOL among adolescents [12, 16, 17, 22, 23, 30, 35–39].

From a health promotion perspective, more knowledge of how sociodemographic variables, self-efficacy, self-esteem, pain, sleep, loneliness and stress are related to adolescents’ HRQOL is needed. In order to gain more knowledge of which of these factors future interventions among school-based populations of adolescents should prioritize, there is a need to simultaneously investigate the impact of these factors on HRQOL. Investigating such associations could inform practice and policy. Furthermore, considering that age is an important predictor of HRQOL, more knowledge about HRQOL in adolescents at a specific age is warranted.

The aim of this study was to assess the associations between sociodemographic variables, self-efficacy, self-esteem, pain, sleep, loneliness, stress, and HRQOL in 14–15-year-old adolescents. Based on theory and earlier research, we hypothesized that there is a positive association between self-efficacy, self-esteem and HRQOL, and that there is a negative association between low SES, female gender, stress, loneliness, pain, higher school absenteeism, lack of sleep and HRQOL.

## Methods

### Sample and data collection

This cross-sectional study was a part of the “Start Young – quality of life and pain in generations” study, which is a longitudinal study that aims to acquire new knowledge about HRQOL and pain in adolescents and their parents, as well as investigate potential family and regional patterns. The present study used data collected at baseline. The Start Young study was conducted in the south-eastern part of Norway, with approximately 1.6 million inhabitants (30% of the total Norwegian population) and an adolescent population (aged 14–15 years) of approximately 37,000. Schools covering 9th grade (aged 14–15 years) in elementary school were stratified according to region, rural and urban districts, and school size. Two schools were randomly selected from each stratum. The schools were each sent a letter of invitation, followed by a telephone call to the school’s principal. Schools that did not choose to participate were replaced by alternative schools selected according to the same criteria. We invited 59 schools and 22 schools agreed to participate. The schools varied in size and localization (from city to suburb) and admitted adolescents with different sociocultural and economic backgrounds. Inclusion criteria for this study were being a student in 9^th^ grade at one of the participating schools, having active informed consent to participate from one parent, giving their own consent to participate and being present at school by the time of data collection. Potential participants in the study were 1663 adolescents in 9th grade from the participating schools of which 967 adolescents were excluded due to lack of active informed consent from parents (n = 872), not giving their own consent to participate (n = 8), technical problems at one school (n = 10) or because they were not present at school by the time of data collection (n = 77). A total of 696 adolescents took part (response rate 41.8%). The response rate varied across schools from 92.1 to 8.6%.

One or two project members visited each school approximately 1 week before data collection to provide the adolescents with verbal and written information about the study. Written information was also distributed to the parents. Active informed consent was obtained from both adolescents and their parents. Data collection was conducted from November 2018 to April 2019. A web-based questionnaire was administered and completed in the classrooms during school hours. One or two project members and a teacher were present to provide assistance when needed. The collected data were stored at a safe data server.

The “Start Young—quality of life and pain in generations” study was reviewed by the Norwegian Centre for Research Data (Ref: 60,981). Necessary approvals were obtained.

## Instruments

### Demographic variables

The first part of the questionnaire included self-reported data on demographic variables such as gender, date of birth, cohabitant status, parental marital status, parents’ birthplace, whether the respondents had moved during the previous 5 years, and school absence.

### Questionnaires

A list of instruments used in this study is presented in Table 1. The internal consistency for multi-item scales was assessed using Cronbach’s alpha [40].

**Table 1:** Overview of instruments used in this study

Factors	Instruments	Number of items	αª
HRQOL	KIDSCREEN-27		
	Physical well-being	5	0.81
	Psychological well-being	7	0.87
	Autonomy and parent relations	7	0.77
	Social support and peers	4	0.78
	School environment	4	0.80
Self-efficacy	Generalized Self‐Efficacy Scale (GSE)	10	0.87
Self-esteem	Rosenberg Self-Esteem scale (RSES)	4	0.79
Pain	Brief Pain Inventory (BPI)		
	Having pain today	1	
	Pain on average	1	
	Pain interference with activity	3	0.81
	Pain interference with emotions	4	0.90
	Lübeck Pain-Screening Questionnaire (LPQ)		
	Pain duration	1	
	Pain frequency	1	
	OTC analgesic questions (derived from SUS)		
	Intake of OTC analgesics during the last 4 weeks	1	
	Frequency of OTC analgesics intake	1	
Sleep	School Sleep Habits Survey		
	Problems with sleepiness	1	
	Frequency of enough sleep	1	
Loneliness	UCLA Loneliness Scale (ULS-8)	8	0.80
Stress	Perceived Stress Questionnaire (PSQ)	30	0.93

OTC, Over-the-counter; SUS, “Pain, youth and self-medication study”

ªCronbach’s α coefficient values in this study

*HRQOL* was measured using the Norwegian version of the KIDSCREEN-27 questionnaire [41, 42]. The KIDSCREEN-27 is a well-validated, short, multidimensional measure of generic HRQOL in children and adolescents organized into five subscales: (1) Physical well-being; (2) Psychological well-being; (3) Autonomy and parent relations; (4) Social support and peers; and (5) School environment [41, 43–45]. The KIDSCREEN instrument is answered on a 5-point Likert scale referring to the last week. The scale indicates either the frequency of certain behaviors or feelings (ranging from “never” to “always”) or the intensity of an attitude (ranging from “not at all” to “extremely”). Rasch scores were computed for each subscale and transformed into t-values as described in the KIDSCREEN manual [45]. The resulting t-values can be used to make comparisons with international t-values based on 14 European countries. These values are normed to a mean of 50 and a standard deviation of 10 [45]. The answers were recoded so that higher values always indicate better HRQOL in the respective subscales. The Norwegian version of the instrument has been demonstrated to be reliable and valid [42].

*Self-efficacy* was measured using the Norwegian version of the Generalized Self‐Efficacy Scale (GSE) that measures optimistic self-beliefs in coping with the demands, tasks, and challenges of life in general [46, 47]. The GSE consists of 10 statements that the respondent rates on a scale from 1 (completely wrong) to 4 (completely right). The respondent’s scores on each item are summed and divided by ten to a GSE score ranging from 1–4, with higher scores indicating higher levels of generalized self‐efficacy. The GSE has been shown reliable and valid [34, 46].

*Self-esteem* was measured using a short version of the Rosenberg Self-Esteem scale (RSES) [48], wherein respondents rate four statements on self-perceptions on a 4-point Likert scale ranging from 1 (strongly disagree) to 4 (strongly agree). The answers were recoded so that higher values always indicate higher levels of self-esteem. The respondent’s scores on each item were summed and divided by 4 into an RSES score ranging from 1–4. The Norwegian four-item version has demonstrated a high degree of correlation (0.95) with the 10-item version [49] and has been used among adolescents [50, 51].

*Pain* was measured using the Brief Pain Inventory (BPI) [52, 53] and selected questions from the Lübeck Pain-Screening Questionnaire (LPQ) [54]. The BPI assesses the subjective intensity of pain and to what extent pain interferes with activity and emotions [52, 53]. Pain interference questions were only administered to those who rated ≥ 1 on the “pain on average” question (indicating that they had pain). The Norwegian BPI has satisfactory psychometric properties [53], and it has previously been used among Norwegian adolescents [55]. Respondents who rated ≥ 1 on the “pain on average” question of the BPI were also administered two follow-up questions from the LPQ referring to pain duration and pain frequency. The LPQ is a structured self-report questionnaire that evaluates the prevalence and consequences of pain [54]. The Norwegian LPQ has satisfactory feasibility, content, and face validity [56]. Finally, two questions derived from the Norwegian “Pain, youth and self-medication study” (SUS) [17, 57] were used to measure the intake of OTC analgesics. The study involved adolescents through a three-step process in the questionnaire development [17, 57]. In our study, the respondents were first asked about OTC analgesic intake during the last 4 weeks. If the answer was “yes,” the respondents were asked about the frequency of intake.

*Sleep* was measured using two questions adapted from the School Sleep Habits Survey [58], one focusing on problems with sleepiness during daily activities and one focusing on frequency of enough sleep. The School Sleep Habits Survey has been widely used for adolescents and has an established validity in comparison to sleep diaries and actigraphy [59]. It has previously been used to assess sleep habits in Norwegian adolescents [60].

*Loneliness* was measured using the eight-item version of the revised UCLA Loneliness Scale (ULS-8) [61]. This instrument is a short version of the widely used 20-item revised UCLA Loneliness Scale (ULS-20) [62] and is considered to be a reliable and adequate measure of loneliness among adolescents [63]. ULS-8 uses a 4-point Likert scale with values ranging from “never” to “always.” The total score ranges from 8 to 32 points, with higher scores suggesting a higher degree of loneliness. Previous studies have recommended ULS-8 as a good substitute for the ULS-20 [61, 63, 64]. The ULS-8 questionnaire was translated into Norwegian as part of this study by using standardized translation procedures according to an international cross-cultural translation manual, which includes forward and backward translations, pre-testing, and cognitive interviews [65]. The reliability of the ULS-8 Norwegian version was verified using the Cronbach’s alpha coefficient, which in this study was 0.80, suggesting good internal consistency for the instrument [40].

*Stress* was measured using the Perceived Stress Questionnaire (PSQ) [66–68]. PSQ is a 30-item questionnaire referring to the last 4 weeks and can be answered with a 4-point rating scale ranging from 1 (almost never) to 4 (almost always). The answers were recoded so that higher values always indicate higher levels of perceived stress. The resulting PSQ total score was linearly transformed between 0 and 1; PSQ = (raw value—30)/90 [66]. Commonly used cutoff levels of stress with respect to the PSQ are low < 0.33, medium 0.33–0.45, moderate 0.45–0.60, and severe > 0.60. The Norwegian version of the instrument has been demonstrated to have good reliability and validity [68, 69].

### Data analyses

Descriptive statistics were calculated for all variables according to gender and presented as means and standard deviation or as median and min/max for continuous variables. Categorical variables were presented as counts and percentages. Associations between pairs of variables were assessed using chi-square test for categorical data. For continuous data, the *t*-test were used for normal distributed data and Mann–Whitney *U* test were used for data that did not follow normal distribution. The study used an electronic survey tool which was designed to consecutively administer the following respective questionnaires. The adolescents were free to end the survey at any time. Most questions included a neutral option. This resulted in all items being answered.

The five KIDSCREEN subscales were selected as the dependent variables for further analyses. The selected covariates were grouped into seven blocks (B1–B7); B1: Sociodemographic variables, B2: Self-efficacy, B3: Self-esteem, B4: Pain on average, B5: Frequency of enough sleep, B6: Loneliness, and B7: Stress. All selected covariates were theoretically known clinically relevant variables reported in previous HRQOL research [8, 22, 23, 25, 30, 33, 38]. To assess possible associations between the covariates in each block and HRQOL, linear regression analyses were fitted separately for each of the five KIDSCREEN subscales. Assumptions for linear regression were checked and fulfilled. Residuals followed normal distribution.

To further assess possible *adjusted* associations with HRQOL, hierarchical regression analyses were conducted (method enter) for the five KIDSCREEN subscales. The covariates were entered into the regression in seven steps based on B1–B7. Seven linear regression models (M1–M7) were fitted for each of the KIDSCREEN subscales by adding variables from a previous model (block) consecutively; later models always included the variables from the previous steps. All tests were two-sided. P-values ≤ 0.01 were considered statistically significant in order to adjust for multiple testing. All analyses were performed using IBM SPSS Statistics (version 26).

## Results

### Characteristics of the sample

Tables 2 and 3 show the sample characteristics for all included variables. In total, 696 participants (57.5% girls) with a median age of 14 years were included in the analyses. In total, more than two thirds of the participants lived with both parents, had parents who were both born in Norway, had parents who were both working, and had 1–2 siblings. There were no statistically significant differences between genders concerning any of the analyzed sociodemographic variables (Table 2).

**Table 2:** Characteristics of the sample (N = 696)

Variable	Total (696)	Boys (n = 296)	Girls (n = 400)	*P* value
Age, mean (SD)	14.09 (0.33)	14.08 (0.36)	14.09 (0.30)	0.905
Adult members of the household, N (%)				0.185
Both parents	508 (73.0)	224 (75.7)	284 (71.0)	
Alternates between two parents	100 (14.4)	45 (15.2)	55 (13.8)	
One parent and one stepparent	20 (2.9)	6 (2.0)	14 (3.5)	
One parent	55 (7.9)	16 (5.4)	39 (9.8)	
Other caregivers	13 (1.9)	5(1.7)	8 (2.0)	
Parents’ marital status, N (%)^a^				0.642
Two parent family	492 (70.7)	212 (71.6)	280 (70.0)	
Single/divorced parent family	204 (29.3)	84 (28.4)	120 (30.0)	
Parents’ birthplace, N (%)				0.267
Both parents born in Norway	551 (79.2)	241 (81.4)	310 (77.5)	
One parent born in Norway	87 (12.5)	30 (10.1)	57 (14.2)	
Both parents born in another country	58 (8.3)	25 (8.4)	33 (8.3)	
Parents’ work status, N (%)				0.013
Both parents working	547 (78.6)	247 (83.4)	300 (75.0)	
One parent working	133 (19.1)	46 (15.5)	87 (21.8)	
No parent working	16 (2.3)	3 (1.0)	13 (3.3)	
Number of siblings, N (%)^b^				0.730
None	30 (4.3)	11 (3.7)	19 (4.8)	
1	263 (37.8)	107 (36.1)	156 (39.0)	
2	244 (35.1)	109 (36.8)	135 (33.8)	
≥ 3	159 (22.8)	69 (36.8)	90 (22.5)	
Moved during the previous 5 years, N (%)^c^				0.027
No	474 (68.1)	215 (72.6)	259 (64.8)	
Yes	222 (31.9)	81 (27.4)	141 (35.3)	
School absence for the previous 3 months, N (%)^d^				0.812
0–4 days	595 (85.5)	255 (86.1)	340 (85.0)	
5–10 days	84 (12.1)	35 (11.8)	49 (12.3)	
> 10 days	17 (2.4)	8 (2.0)	11 (2.8)	

Continuous variables analyzed using independent *t*-test. Categorical variables analyzed using χ^2^-test

SD, standard deviation

^a^The variable was dichotomized as “two parents” (married or cohabiting) or “single parent” (unmarried, divorced, or widowed)

^b^The variable was recoded into four categories: “none,” “1,” “2,” or “ ≥ 3” (3, 4, 5, > 5)

^c^The variable was dichotomized as “yes” (moved 1 time, 2–4 times, ≥ 5 times) or “no.”

^d^The variable was recoded into three categories: “0–4 days” (none, 1–4 days), “5–10 days” (5–7 days, 8–10 days), or “ > 10 days.”

**Table 3:** Descriptive characteristics for HRQOL, self-efficacy, self-esteem, pain, sleep, loneliness, and stress (N = 696)

Variable	Total (n = 696)	Boys (n = 296)	Girls (n = 400)	*P *value
Physical well-being, mean (SD)^a,b^	47.1 (9.3)	49.6 (9.6)	45.2 (8.6)	< 0.001*
Psychological well-being, mean (SD)^a,b^	46.6 (8.4)	49.5 (8.1)	44.4 (8.0)	< 0.001*
Autonomy and parent relations, mean (SD)^a,b^	52.6 (8.7)	53.4 (9.1)	51.9 (8.5)	0.027
Social support and peers, mean (SD)^a,b^	48.4 (8.5)	49.0 (8.5)	48.0 (8.4)	0.130
School environment, mean (SD)^a,b^	48.0 (8.6)	49.6 (9.1)	46.8 (7.9)	< 0.001*
Self-efficacy, mean (SD)^c^	3.1 (0.4)	3.2 (0.4)	3.0 (0.4)	< 0.001*
Self-esteem, mean (SD)^d^	3.1 (0.7)	3.3 (0.6)	2.9 (0.7)	< 0.001*
Having pain today, N (%)				< 0.001*
Yes	200 (28.7)	56 (18.9)	144 (36.0)	
No	496 (71.3)	240 (81.1)	256 (64)	
Pain on average, mean (SD)^e^	2.2 (1.9)	1.6 (1.7)	2.6 (1.9)	< 0.001*
Pain interference with activity, median (min, max)^f, g^	1.3 (0.0, 10.0)	1.0 (0.0, 10.0)	1.3 (0.0, 9.7)	0.372
Pain interference with emotions, median (min, max)^f, g^	1.2 (0.0, 9.7)	0.7 (0.0, 9.2)	1.2 (0.0, 9.7)	< 0.001*
Pain duration, N (%)^f, h^				0.069
Pain ≤ 3 months	335 (63.6)	133 (68.6)	202 (60.3)	
Pain > 3 months	192 (36.4)	61 (31.4)	131 (39.3)	
Pain frequency, N (%)^f, i^				0.146
Seldom	221 (41.9)	92 (47.4)	129 (38.7)	
Sometimes	123 (23.2)	42 (21.6)	81 (24.3)	
Often	183 (34.7)	60 (30.9)	123 (36.9)	
OTC analgesic intake during the last 4 weeks, N (%)				< 0.001*
Yes	342 (49.2)	103 (34.8)	239 (59.9)	
No	353 (50.8)	193 (65.2)	160 (40.1)	
Frequency of OTC analgesic intake, N (%)^j^				0.043
Daily	20 (5.8)	10 (9.7)	10 (4.2)	
Every week, but not daily	52 (15.2)	10 (9.7)	42 (17.6)	
Less often than every week	243 (71.1)	72 (69.9)	171 (71.5)	
No intake during the last 4 weeks	27 (7.9)	11 (10.7)	16 (6.7)	
Problems with sleepiness, N (%)				< 0.001*
No problem at all	280 (40.3)	151 (51.0)	129 (32.3)	
A slight problem	311 (44.7)	120 (40.5)	191 (47.9)	
More than a slight problem	68 (9.8)	17 (5.7)	51 (12.8)	
A big problem	26 (3.7)	7 (2.4)	19 (4.8)	
A very big problem	10 (1.4)	1 (0.3)	9 (2.3)	
Frequency of enough sleep, N (%)				0.002*
Always	59 (8.5)	34 (11.5)	25 (3.6)	
Usually	387 (55.7)	175 (59.1)	212 (53.1)	
Sometimes	177 (25.5)	69 (23.3)	108 (27.1)	
Rarely	63 (9.1)	15 (5.1)	48 (6.9)	
Never	9 (1.3)	3 (1.0)	6 (1.5)	
Loneliness, median (min, max)^k^	13.0 (8.0, 32.0)	12.0 (8.0, 27.0)	13.0 (8.0, 32.0)	< 0.001*
Stress, mean (SD)^l^	0.29 (0.15)	0.24 (0.13)	0.33 (0.16)	< 0.001*

Continuous variables analyzed using independent *t*-test and Mann–Whitney *U* test. Categorical variables analyzed using χ^2^-test

HRQOL, health-related quality of life; OTC, over-the-counter; SD, standard deviation

^a^KIDSCREEN subscales

^b^Rasch scores were computed for each subscale and transformed into t-values with a mean of 50 and an SD of 10. Higher values indicate higher levels of HRQOL

^c^Range 1–4, where higher values indicate higher levels of self-efficacy

^d^Range 1–4, where higher values indicate higher levels of self-esteem

^e^Range 0–10, where 10 indicates pain as bad as you can imagine

^f^N = 527

^g^Range 0–10, where 10 indicates complete interference of pain

^h^The variable was dichotomized as “Pain ≤ 3 months” (only once, < 1 month, 1–3 months) or “Pain > 3 months” (> 3 months, > 6 months, > 12 months)

^i^The variable was recoded into three categories: “seldom” (< once/month, once/month), “sometimes” (2–3 times/month, once/week), or “often” (several times/week, every day)

^j^N = 342

^k^Range 8–32, where higher values indicate higher levels of loneliness

^l^Range 0–1, where higher values indicate higher levels of stress

^*^P ≤ 0.01

Regarding the descriptive characteristics presented in Table 3, several variables significantly differed according to gender. The adolescents generally reported high levels of HRQOL assessed using the KIDSCREEN-27 scores, but girls reported significantly lower levels of HRQOL than boys for the subscales *Physical well-being*, *Psychological well-being,* and *School environment*. Moreover, girls reported significantly lower levels of *Self-efficacy* and *Self-esteem* and higher levels of *Loneliness* and *Stress* than boys. Significantly more girls (36.0%) than boys (18.9%) reported *Having pain today*, and the levels of *Pain on average* and *Pain interference with emotions* were also significantly higher for girls than boys. Further, significantly more girls (59.9%) than boys (34.8%) reported *Intake of OTC analgesics during the last 4 weeks*. Among those who rated ≥ 1 for *Pain on average* (76%), more than one third of the adolescents reported *Pain duration* of more than 3 months (persistent pain) and 34.7% experienced pain often. More than two thirds of the adolescents reported getting enough sleep usually or always. However, significantly more girls than boys reported having *Problems with sleepiness* and less frequently getting enough sleep.

### Crude associations between sociodemographic variables, self-efficacy, self-esteem, pain, sleep, loneliness, stress, and HRQOL examined by linear regression analyses

Multiple linear regression analyses were used to assess possible associations between selected variables and HRQOL. The strength of the associations between the covariates in each block (B1–B7) and the dependent variables (five KIDSCREEN subscales) is further described in terms of the effect sizes (standardized beta) and explained variance (Table 4). The psychosocial variables (*Self-efficacy*, *Self-esteem*, *Loneliness*, and *Stress*) had the largest effects on the outcome for all HRQOL dimensions. *Self-efficacy* and *Self-esteem* were positively associated with HRQOL whereas *Stress* and *Loneliness* were negatively associated. Sociodemographic- (B1), pain- (B4), and sleep-related covariates (B5) were all significantly associated with some of the subscales; however, their effect on the outcome was smaller than that of the psychosocial variables listed above. Being a girl, not living with both parents, not having both parents working, being absent from school more than 0–4 days, having pain, and lacking enough sleep were all independently negatively associated with HRQOL. The explained variance was the highest for *Psychological well-being* (the covariate *Stress* explained 51.8%) and lowest for *Physical well-being* (the covariate *Self-esteem* explained 19.2%).

**Table 4:** Crude associations between sociodemographic variables, self-efficacy, self-esteem, pain, sleep, loneliness, stress, and HRQOL examined by linear regression analyses^a,b,c^ N = 696

	Physical well-being							Psychological well-being						
	B1	B2	B3	B4	B5	B6	B7	B1	B2	B3	B4	B5	B6	B7
Gender (ref = boy)	− 0.21*							− 0.27*						
Adult members of the household (ref = both parents)														
Part-time with each parent	− 0.07							− 0.09						
One parent and one stepparent	− 0.06							− 0.06						
One parent	− 0.08							− 0.12*						
Other caregivers	− 0.02							− 0.08						
Parents’ work status (ref = both parents working)														
One parent working	− 0.10*							− 0.11*						
No parent working	− 0.08							− 0.00						
School absence (ref = 0–4 days)^d^														
5–10 days	− 0.14*							− 0.13*						
> 10 days	− 0.09							− 0.08						
Self-efficacy		0.43*							0.53*					
Self-esteem			0.44*							0.67*				
Pain on average				− 0.29*							− 0.41*			
Frequency of enough sleep (ref = always)														
Usually					− 0.23*							− 0.30*		
Sometimes					− 0.27*							− 0.39*		
Rarely					− 0.31*							− 0.38*		
Never					− 0.18*							− 0.24*		
Loneliness						− 0.37*							− 0.62*	
Stress							− 0.43*							− 0.72*
R^2^ adj	0.106	0.186	0.192	0.086	0.083	0.133	0.187	0.150	0.276	0.449	0.171	0.112	0.390	0.518

	Autonomy and parent relations							Social support and peers						
	B1	B2	B3	B4	B5	B6	B7	B1	B2	B3	B4	B5	B6	B7
Gender (ref = boy)	− 0.06							− 0.05						
Adult members of the household (ref = both parents)														
Part-time with each parent	− 0.06							− 0.01						
One parent and one stepparent	− 0.07							− 0.08						
One parent	− 0.13*							− 0.05						
Other caregivers	− 0.11*							− 0.03						
Parents’ work status (ref = both parents working)														
One parent working	− 0.06							− 0.03						
No parent working	− 0.07							0.05						
School absence (ref = 0–4 days)^d^														
5–10 days	− 0.06							− 0.06						
> 10 days	− 0.02							− 0.05						
Self-efficacy		0.38*							0.32*					
Self-esteem			0.43*							0.35*				
Pain on average				− 0.29*							− 0.14*			
Frequency of enough sleep (ref = always)														
Usually					− 0.15							− 0.22*		
Sometimes					− 0.26*							− 0.24*		
Rarely					− 0.24*							− 0.20*		
Never					− 0.15*							− 0.16*		
Loneliness						− 0.39*							− 0.55*	
Stress							− 0.52*							− 0.39*
R^2^ adj	0.049	0.145	0.181	0.080	0.053	0.153	0.270	0.010	0.104	0.123	0.018	0.032	0.297	0.153

	School environment						
	B1	B2	B3	B4	B5	B6	B7
Gender (ref = boy)	− 0.14*						
Adult members of the household (ref = both parents)							
Part-time with each parent	− 0.03						
One parent and one stepparent	− 0.04						
One parent	− 0.05						
Other caregivers	− 0.02						
Parents’ work status (ref = both parents working)							
One parent working	− 0.07						
No parent working	− 0.05						
School absence (ref = 0–4 days)^d^							
5–10 days	− 0.12*						
> 10 days	− 0.06						
Self-efficacy		0.49*					
Self-esteem			0.51*				
Pain on average				− 0.34*			
Frequency of enough sleep (ref = always)							
Usually					− 0.23*		
Sometimes					− 0.37*		
Rarely					− 0.35*		
Never					− 0.22*		
Loneliness						− 0.40*	
Stress							− 0.56*
R^2^ adj	0.046	0.240	0.262	0.113	0.112	0.162	0.310

^a^Linear regression analyses were performed separately for each of the five KIDSCREEN subscales as the dependent variables.

^b^The independent variables were grouped into seven blocks: B1–B7.

ͨThe strength of the associations is described in terms of standardized regression coefficients and adjusted R^2^.

ͩThe variable was recoded into three categories: “0–4 days” (none, 1–4 days), “5–10 days” (5–7 days, 8–10 days), or “ > 10 days”.

*P ≤ 0.01

### Adjusted associations between sociodemographic variables, self-efficacy, self-esteem, pain, sleep, loneliness, stress, and HRQOL examined by hierarchical regression analyses

Table 5 shows the strength of the adjusted associations from the hierarchical regression analyses between the covariates and the dependent variables described in terms of effect sizes (standardized beta) and explained variance. When all variables were added into model 7, the impact of the sociodemographic variables was diminished compared with the impact from the other covariates. However, *Gender* had the third largest effect size in relation to *Autonomy and parent relations*. Being a girl was positively associated with this KIDSCREEN subscale. The psychosocial variables (*Self-efficacy, Self-esteem, Loneliness,* and *Stress*) revealed the largest effect sizes and also contributed to a considerable increase of the explained variance for all five subscales, suggesting that the psychosocial variables are highly relevant for adolescents’ HRQOL. *Self-efficacy* and *Self-esteem* were positively associated with HRQOL, whereas *Stress* and *Loneliness* were negatively associated. *Pain on average* had a significant negative effect on four KIDSCREEN subscales; however, its effect on the outcome was smaller than that of the psychosocial variables. *Frequency of enough sleep* had the second largest significant effect on *Physical well-being* (lacking enough sleep was negatively associated with HRQOL) but was no longer significantly associated with the other KIDSCREEN subscales when adjusted for available confounders. Given the analyzed variables, the explained variance of model 7 was the highest for *Psychological well-being* (65.8%) and the lowest for *Physical well-being* (30.8%).

**Table 5:** Adjusted associations between sociodemographic variables, self-efficacy, self-esteem, pain, sleep, loneliness, stress, and HRQOL examined by hierarchical regression analyses^a,b,c^ N = 696

	Physical well-being							Psychological well-being						
	M1	M2	M3	M4	M5	M6	M7	M1	M2	M3	M4	M5	M6	M7
Gender (ref = boy)	− 0.21*	− 0.13*	− 0.09*	− 0.07	− 0.06	− 0.07	− 0.06	− 0.27*	− 0.18*	− 0.09*	− 0.06	− 0.06	− 0.07*	− 0.05
Adult members of the household (ref = both parents)														
Part-time with each parent	− 0.07	− 0.05	− 0.04	− 0.04	− 0.04	− 0.04	− 0.03	− 0.09	− 0.06	− 0.04	− 0.04	− 0.04	− 0.04	− 0.02
One parent and one stepparent	− 0.06	− 0.01	− 0.01	− 0.01	− 0.00	0.00	− 0.00	− 0.06	− 0.00	0.01	− 0.00	0.00	0.01	0.01
One parent	− 0.08	− 0.05	− 0.04	− 0.03	− 0.02	− 0.02	− 0.02	− 0.12*	− 0.09*	− 0.06	− 0.05	− 0.05	− 0.05	− 0.04
Other caregivers	− 0.02	− 0.00	0.01	0.02	− 0.02	− 0.03	0.03	− 0.08	− 0.05	− 0.03	− 0.02	− 0.02	− 0.01	− 0.01
Parents’ work status (ref = both parents working)														
One parent working	− 0.10*	− 0.09*	− 0.09*	− 0.08	− 0.09*	− 0.09*	− 0.08	− 0.11*	− 0.10*	− 0.10*	− 0.09*	− 0.10*	− 0.08*	− 0.06*
No parent working	− 0.08	− 0.09*	− 0.09*	− 0.10*	− 0.11*	− 0.10*	− 0.10*	0.00	− 0.01	0.01	− 0.01	− 0.02	− 0.02	− 0.02
School absence (ref = 0–4 days)^d^														
5–10 days	− 0.14*	− 0.12*	− 0.10*	− 0.08	− 0.08	− 0.07	− 0.07	− 0.13*	− 0.11*	− 0.05	− 0.03	− 0.03	− 0.02	− 0.01
> 10 days	− 0.09	− 0.06	− 0.05	− 0.05	− 0.05	− 0.04	− 0.04	− 0.08	− 0.04	− 0.03	− 0.03	− 0.03	− 0.01	0.00
Self-efficacy		0.38*	0.26*	0.25*	0.24*	0.22*	0.21*		0.46*	0.21*	0.20*	0.19*	0.14*	0.11*
Self-esteem			0.24*	0.21*	0.19*	0.15*	0.12*			0.50*	0.46*	0.45*	0.33*	0.21*
Pain on average				− 0.13*	− 0.11*	− 0.10*	− 0.10*				− 0.18*	− 0.17*	− 0.15*	− 0.12*
Frequency of enough sleep (ref = always)														
Usually					− 0.12	− 0.12	− 0.11					− 0.14*	− 0.13*	− 0.11
Sometimes					− 0.18*	− 0.18*	− 0.17*					− 0.12*	− 0.11*	− 0.05
Rarely					− 0.11	− 0.11	− 0.10					− 0.09*	− 0.09*	− 0.03
Never					− 0.08	− 0.08	− 0.07					− 0.08*	− 0.07*	− 0.02
Loneliness						− 0.11*	− 0.09						− 0.32*	− 0.24*
Stress							− 0.07							− 0.31*
R^2^ adj	0.106	0.237	0.275	0.289	0.299	0.307	0.308	0.150	0.343	0.511	0.539	0.545	0.616	0.658

	Autonomy and parent relations							Social support and peers						
	M1	M2	M3	M4	M5	M6	M7	M1	M2	M3	M4	M5	M6	M7
Gender (ref = boy)	− 0.06	0.02	0.07	0.10*	0.10*	0.10*	0.12*	− 0.05	0.02	0.06	0.07	0.07	0.06	0.06
Adult members of the household (ref = both parents)														
Part-time with each parent	− 0.06	− 0.03	− 0.02	− 0.02	− 0.02	− 0.02	− 0.01	− 0.01	0.01	0.02	0.02	0.02	0.02	0.03
One parent and one stepparent	− 0.07	− 0.03	− 0.02	− 0.03	− 0.03	− 0.02	− 0.03	− 0.08	− 0.04	− 0.04	− 0.04	− 0.04	− 0.02	− 0.02
One parent	− 0.13*	− 0.10*	− 0.09	− 0.08	− 0.07	− 0.07	− 0.06	− 0.05	− 0.03	− 0.02	− 0.02	− 0.02	− 0.02	− 0.02
Other caregivers	− 0.11*	− 0.08	− 0.07	− 0.06	− 0.06	− 0.06	− 0.05	− 0.03	− 0.00	0.00	0.01	0.01	0.02	0.02
Parents’ work status (ref = both parents working)														
One parent working	− 0.06*	− 0.06	− 0.06	− 0.05	− 0.06	− 0.04	− 0.03	− 0.03	− 0.03	− 0.03	− 0.03	− 0.03	− 0.00	0.00
No parent working	− 0.07*	− 0.08	− 0.08	− 0.08	− 0.09*	− 0.09*	− 0.09*	0.05	0.04	0.04	0.04	0.03	0.04	0.04
School absence (ref = 0–4 days)^d^														
5–10 days	− 0.06	− 0.05	− 0.01	0.00	0.01	0.01	0.02	− 0.06	− 0.05	− 0.02	− 0.01	− 0.01	0.00	0.00
> 10 days	− 0.02	0.01	0.02	0.02	0.02	0.03	0.04	− 0.05	− 0.02	− 0.01	− 0.01	− 0.02	0.02	0.02
Self-efficacy		0.36*	0.21*	0.20*	0.20*	0.17*	0.14*		0.31*	0.19*	0.19*	0.17*	0.10	0.09
Self-esteem			0.31*	0.27*	0.26*	0.19*	0.06			0.26*	0.25*	0.25*	0.06	0.03
Pain on average				− 0.16*	− 0.15*	− 0.13*	− 0.11*				− 0.03	− 0.03	0.02	0.02
Frequency of enough sleep (ref = always)														
Usually					− 0.05	− 0.04	− 0.01					− 0.13	− 0.12	− 0.11
Sometimes					− 0.08	− 0.08	− 0.01					− 0.10	− 0.08	− 0.07
Rarely					− 0.06	− 0.06	0.00					− 0.05	− 0.07	− 0.05
Never					− 0.05	− 0.04	0.01					− 0.08	− 0.06	− 0.04
Loneliness						− 0.18*	− 0.09						− 0.48*	− 0.45*
Stress							− 0.34*							− 0.10
R^2^ adj	0.049	0.170	0.233	0.255	0.254	0.276	0.324	0.010	0.101	0.144	0.144	0.146	0.306	0.309

	School environment						
	M1	M2	M3	M4	M5	M6	M7
Gender (ref = boy)	− 0.14*	− 0.04	0.02	0.04	0.05	0.04	0.06
Adult members of the household (ref = both parents)							
Part-time with each parent	− 0.03	− 0.01	0.00	0.01	0.01	0.01	0.02
One parent and one stepparent	− 0.04	0.02	0.03	0.01	0.03	0.03	0.03
One parent	− 0.05	− 0.02	− 0.00	0.01	0.02	0.02	0.03
Other caregivers	− 0.02	0.01	0.03	0.03	0.04	0.04	0.05
Parents’ work status (ref = both parents working)							
One parent working	− 0.07	− 0.06	− 0.07	− 0.06	− 0.07	− 0.06	− 0.05
No parent working	− 0.05	− 0.06	− 0.06	− 0.06	− 0.07	− 0.07	− 0.07
School absence (ref = 0–4 days)^d^							
5–10 days	− 0.12*	− 0.10*	− 0.06	− 0.04	− 0.03	− 0.03	− 0.02
> 10 days	− 0.06	− 0.02	− 0.01	− 0.01	− 0.01	− 0.00	0.01
Self-efficacy		0.47*	0.30*	0.30*	0.28*	0.26*	0.24*
Self-esteem			0.34*	0.30*	0.27*	0.23*	0.13*
Pain on average				− 0.18*	− 0.16*	− 0.15*	− 0.13*
Frequency of enough sleep (ref = always)							
Usually					− 0.10	− 0.09	− 0.07
Sometimes					− 0.16*	− 0.16*	− 0.11
Rarely					− 0.13*	− 0.14*	− 0.09
Never					− 0.11*	− 0.10*	− 0.06
Loneliness						− 0.11*	− 0.04
Stress							− 0.26*
R^2^ adj	0.046	0.250	0.327	0.355	0.369	0.377	0.404

^a^Hierarchical regression analyses were performed separately for each of the five KIDSCREEN subscales as dependent variables.

^b^The independent variables were entered into the regression in seven steps, leading to seven linear regression models (M1–M7).

ͨThe strength of the associations is described in terms of standardized regression coefficients and adjusted R^2^

ͩThe variable was recoded into three categories: “0–4 days” (none, 1–4 days), “5–10 days” (5–7 days, 8–10 days), or “ > 10 days”.

*P ≤ 0.01

## Discussion

The aim of this cross-sectional study was to assess possible associations between sociodemographic variables, self-efficacy, self-esteem, pain, sleep, loneliness, stress, and HRQOL in 14–15-year-old adolescents. We found that 14–15-year-old Norwegian adolescents generally report levels of HRQOL that are in line with the results of the European Normdata for KIDSCREEN-27 in 12–18-year old adolescents [45]. However, in line with previous research [21–27], our data confirmed that girls reported lower HRQOL than boys. One of the main findings in this study was that the psychosocial variables (*Self-efficacy*, *Self-esteem*, *Loneliness*, and *Stress*) had the largest effects on the outcome for all HRQOL dimensions both before and after adjustment for selected covariates. Sociodemographic-, pain-, and sleep-related covariates were all significantly associated with some of the subscales; however, their effect on the outcome was smaller than that of the psychosocial variables listed above.

According to our results, stress may be one of the greatest risk factors for adolescents’ HRQOL. Moreover, our findings indicate that this may be especially important to consider in girls, because they reported having medium levels of stress compared with boys who only reported low values of stress. A Norwegian study by Moksnes and colleagues showed that girls had significantly higher mean scores on all stress domains and on emotional states compared with boys, who had higher self-esteem [7]. Additionally, our findings indicate that loneliness should also be considered as an important risk factor due to its large effect size for the KIDSCREEN subscale *Social support and peers*. Adolescence is considered a period of high risk for loneliness [9, 11], and failure to resolve loneliness before the end of adolescence may pose significant concerns for future social relationships and mental health [11].

Our findings highlight the importance of considering high self-efficacy and self-esteem as important protective or resource factors for HRQOL in adolescents, which is in line with previous research [8, 22, 25, 31–34]. Moreover, our results show that in the presence of self-efficacy and self-esteem, the negative effect of stress on HRQOL decreases. Similar to the findings of Freire and Ferreira [22], this indicates that positive psychosocial factors (e.g., self-efficacy and self-esteem) might play a buffer role for negative psychosocial factors (e.g., stress) in adolescents.

This study revealed that many adolescents experienced pain, and girls reported significantly more pain than boys. The intensity of pain reported is not considered high, yet the prevalence is a cause for concern. Even though pain was not found to be a strong explanatory factor for variations in HRQOL, our results support previous research showing that pain is negatively associated with HRQOL in adolescents [16, 23]. Furthermore, we found that approximately half of the adolescents reported intake of OTC analgesics, and more girls than boys reported such intake. Considering the relatively low intensity of pain reported, this might indicate that the adolescents use OTC analgesics for reasons other than only pain relief. Frequent consumption of OTC analgesics may cause health problems such as drug‐induced headache and liver failure [70]; thus, our findings emphasize that the use of OTC analgesics among adolescents should be regarded as a significant health concern. According to Skarstein et al. [70], informing adolescents, parents, and the society in general about how to use OTC analgesics appropriately should be a high priority.

Sleep played an important role for the dimension *Physical well-being* in our study, confirming that sleep is highly important for HRQOL in adolescents [19, 20, 39]. Studies have shown that there are several barriers to healthy sleep among adolescents such as later preferred sleep timing, lower parental supervision of bedtime, longer study time, and early school start time [19, 38, 71]. Thus, prevention of and interventions against sleep problems require collaboration between adolescents, parents, schools, and healthcare professionals [39].

After adjusting for other factors related to HRQOL, gender was statistically significantly associated only with *Autonomy and parent relations*. An interesting finding was also that being a girl was positively associated with this subscale. Possible explanations of our results might be that gender is important to HRQOL, but that part of the differences between boys and girls in HRQOL can be explained by psychosocial factors. Also, our results show that girls scored significantly worse on pain- and sleep related factors which also are associated with HRQOL.

### Strengths and limitations

The main strengths of this study include the relatively large sample of 14–15-year-old adolescents in a school-based setting, and that the selected analyzed variables were all assessed with well-validated instruments. The results of this study may be regarded as representative of adolescents in the south-eastern part of Norway; however, we do not know whether they can be generalized to the rest of Norway. Nevertheless, the school system in Norway is fairly homogeneous considering that the majority of adolescents are attending public schools [72], indicating that the findings should be similar for the same age group in other Norwegian regions. However, more than two thirds of the participants lived with both parents, had parents that were both born in Norway and had parents that were both working, indicating that the results may not be representative for adolescents that come from families with lower SES. This should be taken into consideration when interpreting our results.

This was a cross-sectional study, which makes it impossible to determine causal inference. Another limitation is linked to non-participation. Overall response rate was only 41.8%, and we do not have information to assess whether the participants and nonparticipants differed in any respect. Still, it seems plausible that the use of active consent from parents may have resulted in a biased sample, considering the low response rate. Several adolescents said that they wanted to participate but had forgotten to ask their parents for consent or had forgotten to bring their parents’ consent form at the time of data collection. We cannot assume if there were any differences between those who had the written consent or not. We may only speculate that parents with high education were more likely to deliver informed consent. However, due to General Data Protection Regulation laws we were not allowed to ask non-responders anything. Furthermore, we did not control for other possible confounders such as bullying and digital technology use. Hence, controlling for other confounders are recommended in future studies.

### Clinical implications and future research

Overall, this study contributes to more knowledge of how sociodemographic variables, self-efficacy, self-esteem, pain, sleep, loneliness and stress are related to HRQOL in 14–15-year-old adolescents. To promote HRQOL among adolescents, we suggest that future interventions should prioritize their attention towards psychosocial factors. Interventions aimed at preventing negative psychosocial factors (e.g., stress), might be performed through the promotion of self-efficacy and self-esteem. Moreover, our findings indicate that to develop efficient HRQOL-promoting interventions, future studies should consider possible gender differences within factors that are associated with HRQOL. We encourage future research to use longitudinal designs to explore our findings more thoroughly. Considering that adolescents spend most of their time in school, we suggest the school setting as an important arena for HRQOL-promoting interventions.

## Conclusions

In this cross-sectional study among 14–15-year-old adolescents in a school-based setting, we found that psychosocial factors (self-efficacy, self-esteem, loneliness, and stress) are more strongly associated with HRQOL, than sociodemographic-, pain-, and sleep-related factors. Our findings indicate that positive psychosocial factors such as self-efficacy and self-esteem might play a buffer role for negative psychosocial factors (e.g., stress) in adolescents. Furthermore, our results showed that girls score significantly worse on HRQOL, self-efficacy, self-esteem, pain, sleep, loneliness, and stress compared with boys. To improve HRQOL in school-based populations of adolescents, we suggest that future interventions should prioritize their attention towards psychosocial factors, especially towards a strengthening of the adolescents’ self-efficacy and self-esteem. We recommend gender-specific interventions.

## Data Availability

The datasets used and/or analyzed during the current study are not publicly available due to General Data Protection Regulation laws but are available from the corresponding author on reasonable request and with permission from the Norwegian Centre for Research Data.

## Abbreviations

BPI: Brief Pain Inventory
GSE: General self-efficacy scale
HRQOL: Health-related quality of life
LPQ: Lübeck Pain-Screening Questionnaire
OTC analgesics: Over-the-counter analgesics
PSQ: Perceived Stress Questionnaire
QOL: Quality of life
RSES: Rosenberg Self-Esteem scale
SES: Socioeconomic status
SUS: Pain, youth and self-medication study
ULS: UCLA Loneliness Scale
WHO: World Health Organization
